# CT volumetry performs better than nuclear renography in predicting estimated renal function one year after living donation

**DOI:** 10.1007/s11255-022-03441-9

**Published:** 2022-12-24

**Authors:** Manuela Almeida, Pedro R. Pereira, Miguel Ramos, Diogo Carneiro, Mariana Mandaleno, Filipa Silva, Sofia Pedroso, Manuela França, La Salete Martins, Jorge Malheiro

**Affiliations:** 1 Department of Nephrology, Centro Hospitalar Universitário do Porto (CHUPorto), Largo Professor Abel Salazar, 4099-001 Porto, Portugal; 2 School of Medicine and Biomedical Sciences, UMIB, Unit for Multidisciplinary Research in Biomedicine, ICBAS, University of Porto, Porto, Portugal; 3 ITR, Laboratory for Integrative and Translational Research in Population Health, Porto, Portugal; 4 Department of Nephrology, Centro Hospitalar de Trás-os-Montes e Alto Douro (CHTMAD), Vila Real, Portugal; 5 Department of Urology, Centro Hospitalar Universitário do Porto (CHUPorto), 4099-001 Porto, Portugal; 6 Department of Radiology, Centro Hospitalar Universitário do Porto (CHUPorto), 4099-001 Porto, Portugal

**Keywords:** Split renal function, Split renal volume, CT scan volumetry, Nuclear renography

## Abstract

The evaluation of split renal function (SRF) is a critical issue in living kidney donations and can be evaluated using nuclear renography (NR) or computerized tomography (CT), with unclear comparative advantages. We conducted this retrospective study in 193 donors to examine the correlation of SRF assessed by NR and CT volumetry and compared their ability to predict remaining donor renal function at 1 year, through multiple approaches. A weak correlation between imaging techniques for evaluating the percentage of the remaining kidney volume was found in the global cohort, with an *R*^2^ = 0.15. However, the Bland–Altman plot showed an acceptable agreement (95% of the difference between techniques falling within − 8.51 to 6.11%). The predicted and observed eGFR one year after donation were calculated using the CKD-EPI, and CG/BSA equations. CT volume showed a better correlation than NR for both formulas (adjusted *R*^2^ of 0.42. and 0.61 vs 0.37 and 0.61 for CKD-EPI and CG/ BSA equations, respectively). In non-nested modeling tests, CT volumetry was significantly superior to NR for both equations. CT volumetry performed better than NR in predicting the estimated renal function of living donors at 1-year, independently from the eGFR equation.

**Supplementary Information:**

The online version contains supplementary material available at 10.1007/s11255-022-03441-9.

## Introduction

End-stage renal disease (ESRD) is a global health problem [1]. Living donor kidney transplants represent the best treatment for ESRD, with increased graft and patient survival compared to deceased kidney transplants [2]. Living donor nephrectomy is considered a safe procedure with low risk, and long-term follow-up data of donors are reassuring [3, 4]. However, two recent landmark studies showed an increased risk of ESRD in kidney donors compared with matched healthy non-donors [5, 6]. Additionally, the increasing shortage of organs for transplantation led to the acceptance of donors with minor abnormalities, usually referred to as “complex kidney donors” [7]. The absence of high-quality studies with long-term follow-up makes the safety of kidney donors an important issue. Defining precise metrics for accepting or discarding a given donor is currently a significant challenge for the transplant community.

Evaluation of living kidney donors is a complex and multidisciplinary task. Laboratory and imaging techniques complement medical and psychosocial assessments. This is a longstanding process [8], and most potential living donors end the process without becoming actual donors. Simplifying living donor evaluation and eliminating unnecessary examinations can increase its success if it does not compromise donor safety or the quality of the global process.

A particular focus of this process is the evaluation of renal function to predict the remaining renal function in the donor and estimated graft function in the recipient. Although the urinary clearance of an “ideal” filtration marker is considered the “gold standard” for the measurement of glomerular filtration rate (GFR), it is unsuitable for clinical practice [9]. Most Eurotransplant centers use creatinine-clearance (64%) to measure total renal function and radioisotope methods (82%) to assess the split renal function (SRF) [10]. In addition, radiological techniques, such as computed tomography (CT) and ultrasound, are used to analyze kidney anatomy. If there is a significant asymmetry, usually considered > 10% of SRF difference between both kidneys, the kidney with the lower function should be preferred for donation, even if it has anatomical variability [9].

Kidney volume has been proven to be a surrogate marker of nephron mass and renal function in living donors [11]. Evaluating renal volume using CT volumetry is routine in several transplant centers [2]. A recent meta-analysis suggested that split renal volume has the potential to replace split renal function in some candidates, eliminating this test from the evaluation process [12], and current evidence suggests [13] that CT volumetry should be preferred mainly when discordance is found between the two imaging modalities. However, it is uncertain whether it can do so reliably and routinely across transplant centers, and global recommendations remain unclear.

We reviewed the practice at our center with the hypothesis that relative kidney volume determined by CT can be used as a substitute for SRF, as determined by nuclear renography, eliminating the need for additional tests in some potential donors. Hence, we assessed the correlation between imaging techniques in the evaluation of SRF and compared their ability to predict remaining kidney function following living donor nephrectomy.

## Materials and methods

We retrospectively reviewed the clinical data of all the donors who underwent nephrectomy for living donor kidney transplantation at our institution between January 2008 and December 2017 (*n* = 210). After excluding 17 donors (10 without CT scan images available, one without nuclear renography evaluation, and another 6 in whom 1 year post-donation eGFR was lacking), the remaining 193 recipients defined our study cohort. The Institutional Review Board at Centro Hospitalar Universitário Porto (CHUPorto) approved this retrospective observational study, conducted according to the Helsinki Declaration.

Following international guidelines, all donors were subjected to a standard evaluation protocol. Baseline demographic, anthropomorphic, analytical, and clinical data were collected from the living kidney donors. Serum creatinine-based Chronic Kidney Disease Epidemiology Collaboration (CKD-EPI), and Cockcroft-Gault adjusted for body surface area (CG-BSA) equations were used to evaluate eGFR before and one year after donation. Creatinine clearance (CrCl) in a 24 h pre-donation urinary sample was available for all donors.

SRF was assessed by nuclear renography with technetium-labeled diethylenetriamine-pentaacetate (Tc99m-DTPA) using a simplified, standardized protocol with dynamic image acquisition in the supine position according to international guidelines [14]. Adequate hydration was ensured in all the donors. The clearance of radionuclides was used as a measure of GFR. Split renal function was evaluated based on each kidney's contribution to global renal function.

For anatomical evaluation, all living donors underwent one of two multidetector-row CT scans available at our institution (64-detector GE VCT LightSpeed” or 16-detector GE BrightSpeed). Images were obtained before and after contrast to evaluate the nephrographic and excretory phases of enhancement. Kidney volumes were retrospectively assessed using CT scans with the same image-acquisition protocols. Volumes were measured using the voxel counting technique (the sum resulting from tracing the renal contours in sequential 2.5 mm transversal CT nephrographic images, excluding the renal sinus area) using the Osirix software (Pixmeo Sarl, Geneva, Switzerland). Both kidneys were evaluated separately. One surgeon performed all the evaluations. The SRF was defined as the percentage of the total renal volume for each kidney.

The preoperative eGFR from each equation was multiplied by the kidney split renal function percentage for each imaging technique to determine the predicted remaining renal function after nephrectomy. The estimated GFR at one year was also calculated using the CG-BSA, and CKD-EPI equations.

Procurement of the left kidney was preferred to balance the lengths of the renal vein and artery and facilitate anastomoses on the corresponding external iliac vessels, except in rare cases of anatomic variations or significant left kidney SRF.

Continuous data were described using mean and standard deviation (SD) or median (interquartile range [IQR]), and categorical data were expressed as numbers (and percentages) as appropriate.

First, we calculated the difference between the right and left kidneys using each imaging technique. Cohen’s kappa test was then applied to measure agreement between the methods. Full agreement was defined as a difference between − 5 to + 5% in both techniques. We weighed the kappa test to deal with disagreement by assigning less weight to agreement categories that were further apart (i.e., giving more weight to the agreement, less weight to the partial agreement, and the smallest weight to no agreement) between techniques. A kappa score of 1 denotes perfect agreement, whereas a kappa score of 0 denotes chance agreement. The difference between the observed and chance agreements was considered significant if the p-value was less than 0.05.

We then analyzed the correlation (by linear regression) and agreement (by Bland–Altman plot) between the remaining kidneys’ CT volumetry and nuclear renography-based SRF. Moreover, univariate and multivariable linear regression explored the correlation of predicted renal function according to each imaging modality and eGFR equation and observed renal donor function at 1 year post-donation. Multivariate analyses were adjusted for age, sex, laterality of the remaining kidney, and body mass index (BMI).

We subsequently performed non-nested modeling to compare the ability of each imaging technique to predict the residual donor kidney function. Non-nested tests assume that one model fits the observed data more closely. A significant *P*-value of a non-nested test indicates that the alternative model has a better fit. R2 compared non-nested models: root mean square error, J-test, and Cox–Pesaran test. The *R*^2^ values were adjusted for degrees of freedom, in that they accounted for the number of explanatory terms in the model.

Finally, a sensitivity analysis was performed considering the agreement between the SRF techniques for the remaining kidneys, which allowed the definition of three groups (agreement, higher SRF of the remaining kidney by nuclear renography, and higher SRF of the remaining kidney by CT scan). These were included in three distinct multivariable linear regression models that determined their correlation with renal donor function at 1 year post-donation per eGFR equation evaluated.

A 2-sided *P*-value < 0.05 was considered as statistically significant. Statistical calculations were performed using STATA/MP, version 15.1 (Stata Corp, College Station, TX, USA).

## Results

The baseline characteristics of the patients are shown in Table 1. The median age of donors was 48.9 (40.6–56.2) years, and most were female (74%). The median BMI was 25.2 (22.7–28.0). The mean pre-donation eGFR was 100.4 ± 13.9 ml/min/1.73 m^2^ by CKD-EPI, and 106.7 ± 21.9 ml/min/1.73 m^2^ by CG BSA adjusted. Pre-donation 24 h creatinine clearance was 128.3 ± 29.5 ml/min/1.73 m^2^, higher than eGFR by any of the formulas, although the difference was lowest with eGFR by the CG BSA-adjusted equation. In most cases, the left kidney was donated (*n* = 158, 82%).

**Table 1:** Baseline characteristics of living kidney donors

	*N* = 193
Age, median (IQR)	48.9 (40.6–56.2)
Sex F:M, *n* (%)	142 (74):51 (26)
BMI, median (IQR)	25.2 (22.7–28.0)
Estimated pre-donation renal function (ml/min/1.73 m^2^), mean ± SD	
CKD-EPI	100.4 ± 13.9
CG (BSA adjusted)	106.7 ± 21.9
Split function of remaining kidney by CT (%), mean ± SD	49.9 ± 3.3
Split function of remaining kidney by nuclear renography: (%), mean ± SD	51.1 ± 3.4
Predicted post-donation renal function (ml/min/1.73 m^2^), mean ± SD	
CKD-EPI CT	50.0 ± 7.1
CKD-EPI NR	51.2 ± 7.5
CG CT	53.2 ± 11.3
CG NR	54.4 ± 11.0
Pre-donation 24 h creatinine clearance (ml/min/1.73 m^2^), mean ± SD	128.3 ± 29.5
Predicted post-donation 24 h creatinine clearance (ml/min/1.73 m^2^), mean ± SD	
CT	64.0 ± 14.9
NR	65.4 ± 15.0
Left kidney donated, *n* (%)	158 (82)
Estimated donor renal function at 12-months (ml/min/1.73 m^2^), mean ± SD	
CKD-EPI	71.2 ± 14.5
CG (BSA adjusted)	74.7 ± 15.3

Split renal function of the remaining kidney by CT-scan was 49.9 ± 3.3% and by nuclear renography was 51.1 ± 3.4%. Predicted and estimated post-donation renal function at 12 months after donation are depicted in Table 1, according to the imaging technique and the formula used to evaluate eGFR. The eGFR after donor nephrectomy was higher than the predicted renal function measured for every imaging technique and the eGFR equation.

The SRF agreement between CT volumetry and nuclear renography is presented in Table 2. Considering the difference in split function between right and left kidneys between − 5 and  + 5%, we observed complete agreement between techniques in 106 donors (55%). In the other donors, we found disagreement between methods with a higher split function (R-L) by nuclear renography in 57 donors and CT volumetry in 30 donors. The kappa test showed a weighted agreement of 75%, expected agreement of 68%, and kappa of 0.259.

**Table 2:** Agreement between techniques of SRF

Nuclear renography: difference in split function between right and left kidneys (R–L)	CT volumetry: difference in split function between right and left kidneys (R–L)			
	< − 5%	− 5 to 5%	> 5%	Total
< − 5%	16	19	2	37
− 5 to 5%	28	72	9	109
> 5%	8	21	18	47
Total	52	112	29	193

Observed full agreement: 106 (55%)

Reclassification for a more positive difference in split function (R-L) by CT volumetry: 30 (16%)

Reclassification of a more positive difference in split function (R-L) by nuclear renography: 57 (30%)

Weighted agreement: 75%, Expected agreement: 66%, Kappa: 0.259

When right (Supplemental Table 1) and left (Supplemental Table 2) remaining kidneys were assessed separately, the observed complete agreement was, respectively, 57% (kappa 0.272) and 46% (Kappa 0.127). The worst agreement was found in the smaller group of donors with the remaining left kidney. Procurement of the left kidney was preferred, except in rare cases of anatomic variations or significant left kidney SRF. This represented a small proportion of our donors. Considering the influence of the depth of the kidneys on the variance of nuclear scintigraphy results and the fact that a lower number of cases implies more heterogeneity, we could assume those effects on the results.

**Fig. 1:**
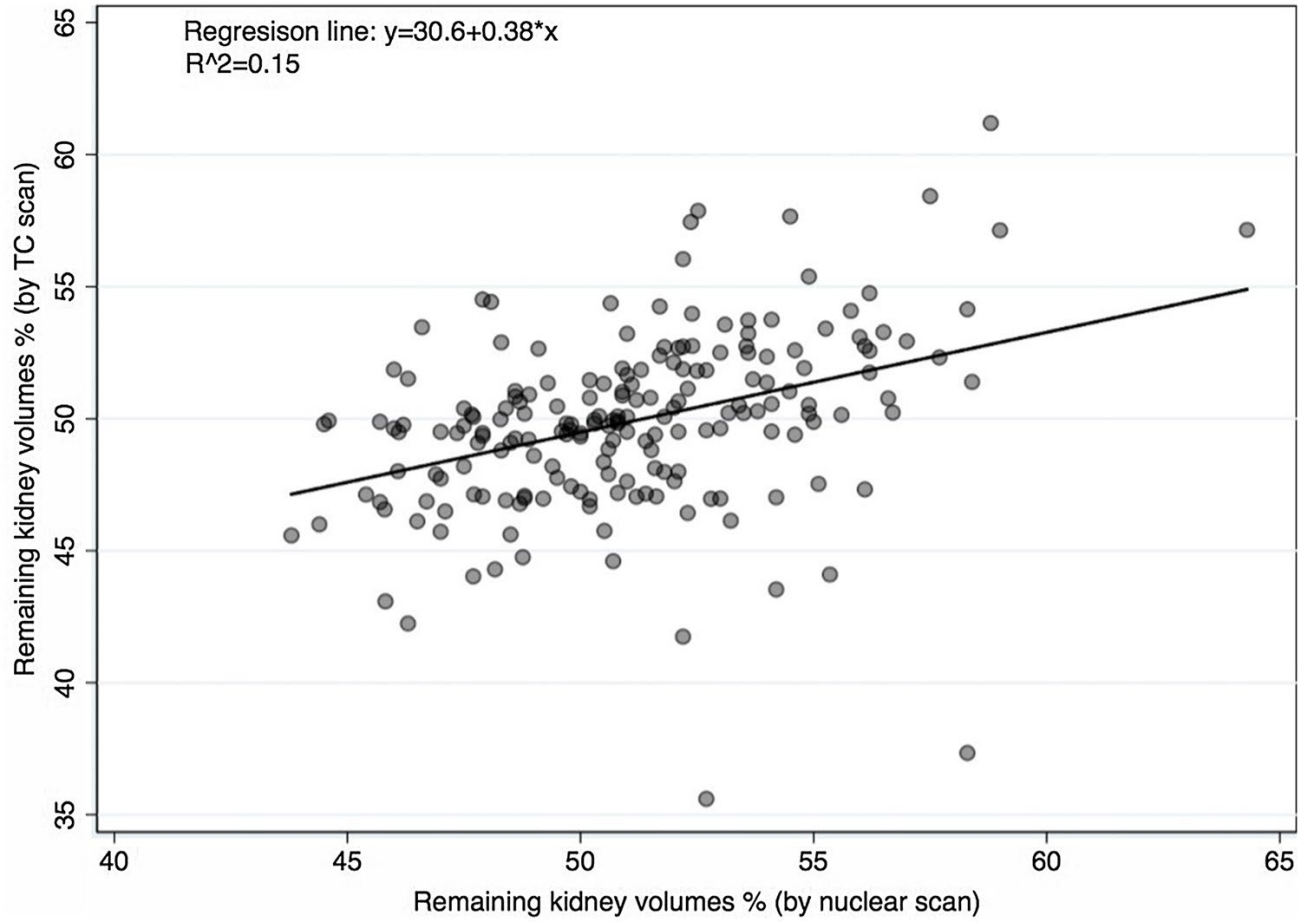
Correlation between CT volumetry and nuclear renography for the determination of the remaining kidney volumes

The linear regression analysis has shown a weak correlation between imaging techniques for evaluating the percentage of remaining kidney volume with an *R*^2^ = 0.15 for the global cohort (Fig. 1).

**Fig. 2:**
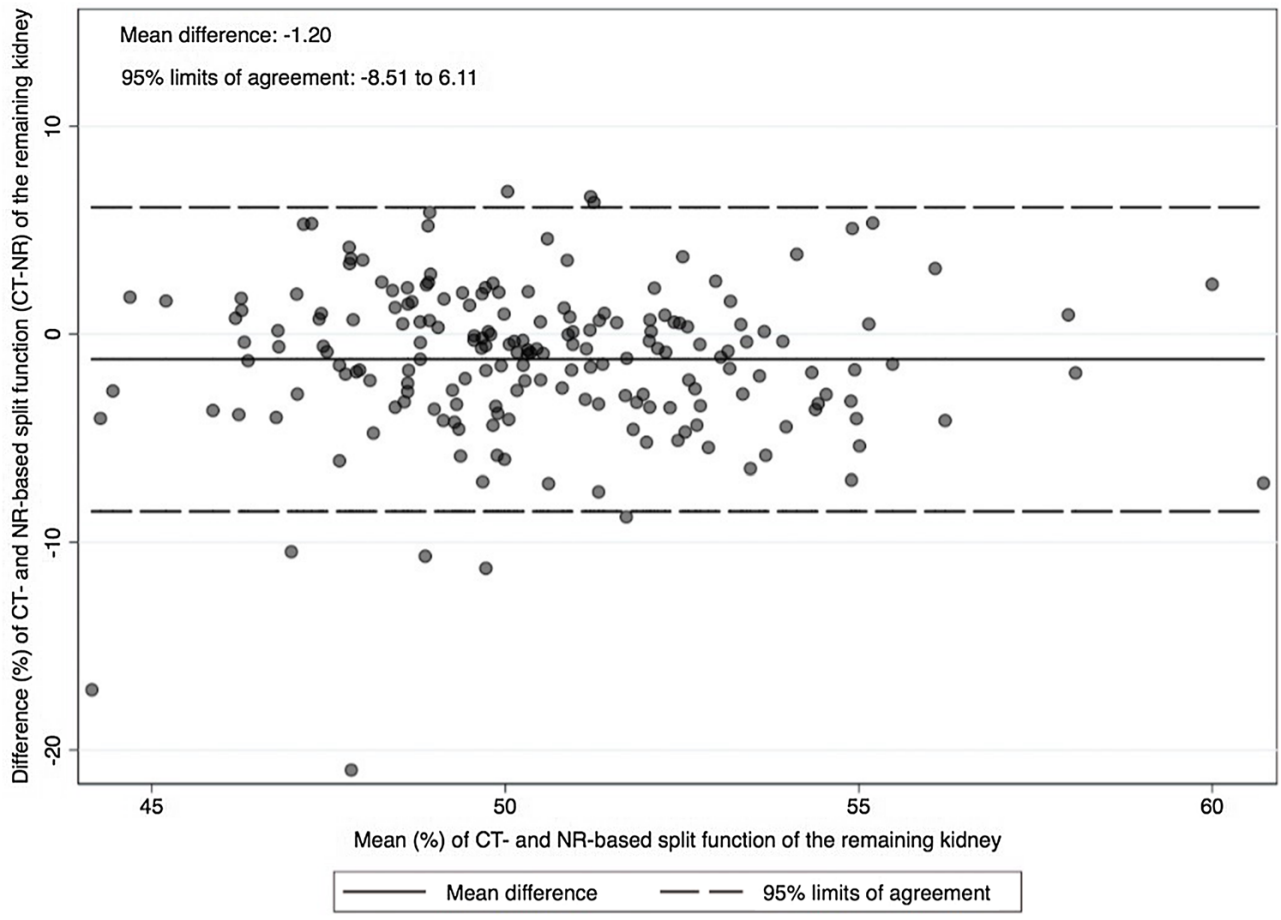
The Bland–Altman plots with percentage SRF analysis between CT volumetry versus nuclear renography

The Bland–Altman plot was used to assess the agreement between the two different clinical measures, assuming neither was perfect: CT-based vs. nuclear renography-based determinations of SRF of the remaining kidney (Fig. 2). This analysis suggested a good clinical agreement, with 95% of the difference between techniques falling within − 8.51 to 6.11%. The mean difference was − 1.2%. On average, nuclear renography measures 1.2% more than CT scan for SRF of the remaining kidney, according to the use of nuclear renography to decide on the nephrectomy side.

**Fig. 3:**
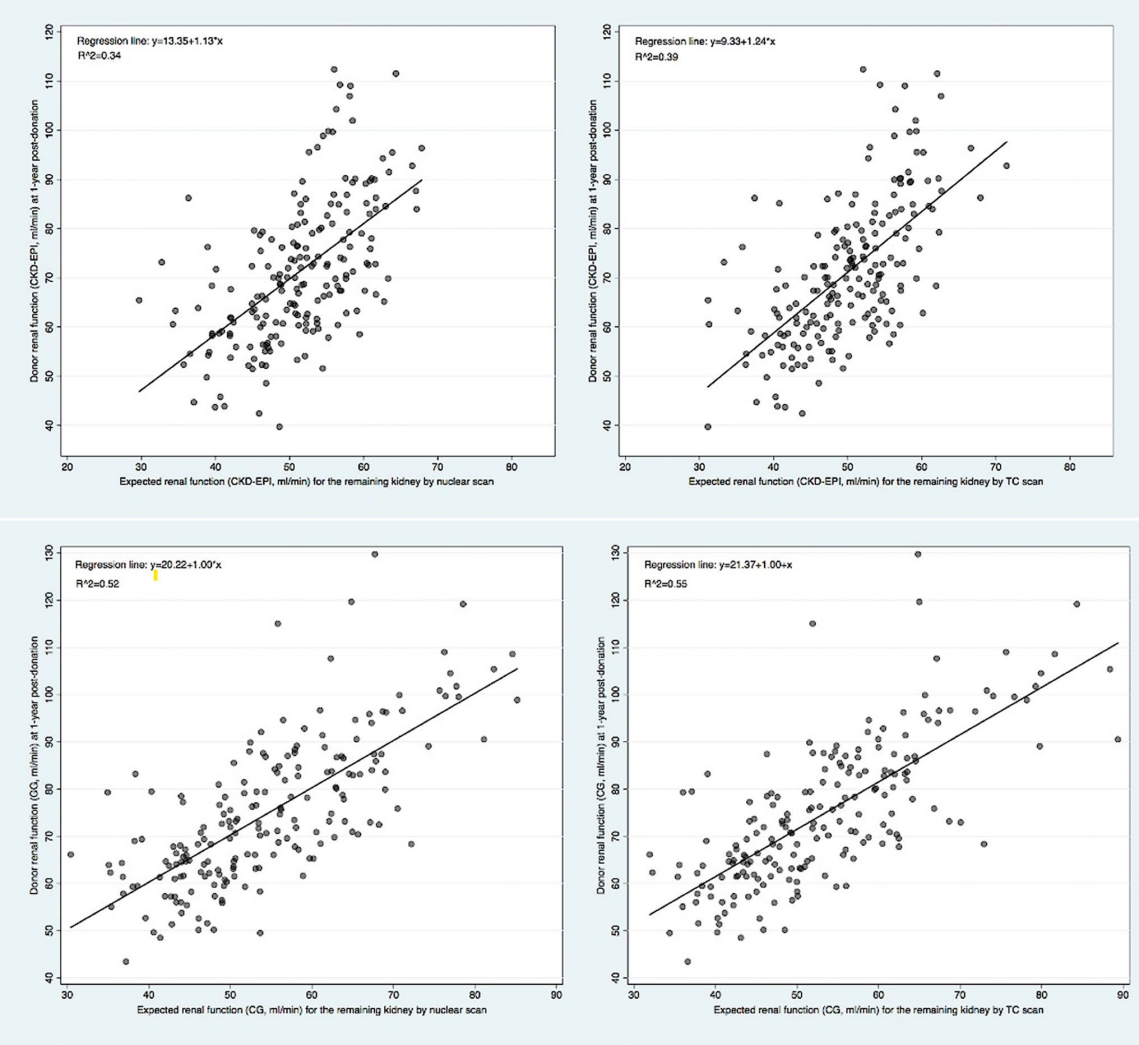
Correlation between expected and observed eGFR one year after donation for nuclear renography (left column images) and CT volumetry

The predicted and observed eGFR were calculated using the CKD-EPI, and CG-BSA equations. The univariate linear regressions of the predicted and observed eGFR according to the imaging technique and eGFR equation are shown in Fig. 3.

Multivariate linear regression analysis of the predicted and observed eGFR was performed for both imaging techniques, adjusted for age, sex, laterality of the remaining kidney, and BMI (Table 3).

**Table 3:** Multivariable linear regression analysis of renal donor function at 1-year post-donation, according to each imaging modality prediction

Formula used	Imaging modality	Coefficient of renal function for every 10 ml/min predicted (95% CI)	*P*	Adjusted *R*^2^	rMSE
CKD-EPI	Nuclear scan	9.56 (7.08–12.05)	< 0.001	0.37	11.52
	CT volume	10.98 (8.51–13.45)	< 0.001	0.42	11.09
CG	Nuclear scan	7.52(6.01–9.02)	< 0.001	0.60	9.71
	CT volume	7.71 (6.25–9.17)	< 0.001	0.61	9.51

Adjusted for age, sex, laterality of remaining kidney, and BMI

The results of both imaging techniques correlated with the eGFR at one year. However, CT volume SRF showed a better correlation than nuclear renography for both formulas tested (adjusted *R*^2^ of 0.42, and 0.61 vs. 0.37, and 0.61 for CKD-EPI, and CG-BSA equations). Interestingly, the best correlation was found with eGFR using the CG-BSA adjusted formula.

We then used non-nested modeling to compare imaging techniques to predict renal donor function one year after donation. Competing models: 1-nuclear renography and two CT-scan volumetry were considered (Supplemental Table 3). The null hypothesis of Model 1 was rejected in all tests (*P* < 0.05), but we failed to reject the null hypothesis that Model 2 was superior (*p* > 0.05). We concluded that CT volumetry was superior to nuclear renography in predicting donor renal function at 1-year post-donation for both equations (CKD-Epi equation: J-test *P*-value < 0.001, Cox-Pesaran Test *P* value < 0.01; CG-BSA: J-test *P* value 0.002, Cox-Pesaran Test *P*-value < 0.01).

Additionally, we performed a sensitivity multivariable linear regression analysis of renal donor function at one-year post-donation, according to the agreement between the SRF techniques of the remaining kidneys (Table 4). The group with complete agreement was considered as the reference in this analysis. Models 1 and 3 showed that a higher SRF predicted by nuclear renography was significantly associated with a lower one-year observed remaining function. In contrast, in Model 2, this observation was only significant for the CKD-EPI-based eGFR, and trended in the same direction for CG-BSA equation.

**Table 4:** Multivariable linear regression analysis of renal donor function at 1-year post-donation, according to split function agreement between CT volumetry and nuclear renography and remaining kidney—model 1, 2 and 3

Formula used	Group	Coefficient (95% CI)	*P*
*Model 1*			
CKD-EPI	Full agreement	Ref	
	Higher SF by CT	− 3.10 (− 8.60 to 2.39)	0.266
	Higher SF NR	− 5.32 (− 9.52 to − 1.12)	0.013
CG	Full agreement	Ref	
	Higher SF by CT	− 2.78 (− 7.76 to 2.21)	0.273
	Higher SF NR	− 4.86 (− 8.67 to − 1.05)	0.013
*Model 2*			
Formula used	Group	Coefficient (95% CI)	*P*
CKD-EPI	Full agreement	Ref	
	Higher SF by CT	− 0.06 (− 4.66 to 4.54)	0.979
	Higher SF NR	− 4.25 (− 7.74 to − 0.76)	0.017
CG	Full agreement	Ref	
	Higher SF by CT	− 1.24 (− 5.23 to 2.75)	0.539
	Higher SF NR	− 3.02 (− 6.08 to 0.04)	0.053
*Model 3*			
Formula used	Group*estimated pre-donation renal function (1-way interaction)	Coefficient (95% CI)	P*
CKD-EPI	Full agreement*eGFR pre-donation	0.63 (0.50–0.77)	Ref
	Higher SF by CT*eGFR pre-donation	0.63 (0.48–0.77)	0.710
	Higher SF NR*eGFR pre-donation	0.59 (0.45–0.73)	0.010
CG	Full agreement*eGFR pre-donation	0.40 (0.33–0.48)	Ref
	Higher SF by CT*eGFR pre-donation	0.38 (0.30–0.47)	0.284
	Higher SF NR*eGFR pre-donation	0.37 (0.29–0.45)	0.025

Model 1 covariates: age, sex, laterality of remaining kidney, and BMI

Model 2 covariates: age, sex, laterality of remaining kidney, BMI, and estimated pre-donation renal function

Model 3 covariates: age, sex, laterality of remaining kidney, BMI

*P value from the Wald test comparing the equality of each coefficient with the reference value

## Discussion

The current protocol for renal imaging evaluation of potential living donors at our institution includes a CT scan for anatomical assessment and nuclear renography to assess SRF for all candidates. The decision of the kidney to donate and accept the donor depends on these results, with the primary goal of protecting and benefiting the donor. Thus, the correct identification of the SRF is critical. Our study supports using relative kidney volume determined by CT as a surrogate for SRF, as determined by nuclear renography, potentially eliminating the need for additional tests in some donors [10]. CT volumetry correlated better with one-year remaining kidney eGFR, mainly through the CG-BSA equation (Fig. 3). In multivariate analysis, CT volumetry performed better as a predictor of one-year donor eGFR (Table 3) and performed better in non-nested modeling, comparing both imaging techniques (Supplemental Table3).

Furthermore, in the multivariate sensitivity analysis, our previous data were supported; in the groups without agreement between methods, a higher remaining kidney SRF by nuclear renography was associated with worse one-year observed remaining kidney function (Table 4). Our results are in line with recently published work from Eum et al. [13] that compared concordant and discordant subgroups according to CT volumetry and nuclear renography results and found a better predictive value of kidney function at several points after donation for CT volumetry. Donor safety is paramount during this major surgery.

Nuclear renography is a method for determining the SRF at most centers, although it is time-consuming and exposes the donor to radioisotopes. Moreover, renography results have been shown to have a relatively wide range of average values because of anatomical variations in kidney location, patient body type, state of diuresis, and operator-dependent variability [15, 16]. Geometric mean images from combined anterior and posterior views offer a more accurate and precise split renal function assessment [17], but these techniques are not widely used. Finally, the radionuclide trace used is variable; Tc-99 m-mercapto-acetyltriglycine (MAG3) and Tc-99 m-DTPA scans are often used based on in-center availability and experience.

The iodinated contrast material used for the CT scan is excreted by the kidneys, mainly by passive glomerular filtration. The plasma clearance of contrast has been used to evaluate renal function [18]. More recent studies evaluated the use of CT scan calculated split renal volume (SRV) as a measure of SRF, considering kidney volume as a surrogate marker of nephron mass and renal function in living donors. Most studies have shown a strong correlation between these two imaging techniques. Several methods have been described for volumetric evaluation. Total parenchymal and renal cortex volume evaluations have both been described. In some studies, only selected donors with asymmetric image evaluation by CT scan or renal ultrasonography were evaluated by nuclear renography [10, 12, 19–21]. Halleck et al. [10], in a retrospective evaluation of 167 consecutive living kidney donors, [10] found a strong correlation between CT-measured split cortex volume (3D reconstruction volume calculation) and MAG3-measured split renal function (*R* = 0.93; *P* < 0.001). The correlation between SRV and remaining renal function (CG) was also significant (*R* = 0.83; *P* < 0.001), as was the case for the recipient (*R* = 0.75; *P* < 0.001) [10].

Wahba et al. [19] evaluated three CT volumetry techniques (modified ellipsoid volume [MELV], smart region of interest [ROI] volume, and renal cortex volume [RCV]) in 101 LKD to calculate the SRF and compared the results with the MAG3 scan. RCV was determined to be the most accurate technique for pre-donation SRF and allowed reliable prediction of the postoperative renal function of the remaining kidney in the donor.

Mitsui et al. [20] assessed the renal cortex and parenchymal volume in 34 Japanese donors using automated CT volumetry and found a strong correlation with MAG3 measured SRF (cortex, *R* = 0.921; parenchyma, *R* = 0.942). Additionally, eGFR measured at any point after donation (3, 6, and 12 months) correlated with SRF measured by MAG3 and cortex or parenchymal volume. They suggested that parenchymal volumetry might sufficiently predict the eGFR after donation in healthy individuals. For technical reasons, we could not evaluate the cortical volume of our donors, but it should not preclude our results. Overall, these results agree with the data presented herein.

Our study found only a weak correlation of both imaging techniques for evaluating the percentage of remaining kidney volume with an *R*^2^ = 0,15 for the global cohort (Fig. 1). We did not find an apparent reason for the low correlation, but we hypothesized that the technical aspects of imaging acquisition, processing, and interpretation could explain some differences. The volumes of the kidneys (all parenchyma) were evaluated retrospectively, through the voxel counting technique, semi-automatically, using volume analyzer software, and the same surgeon performed all volume measures. In contrast, different operators have conducted nuclear renography studies over a long period. Habbous et al. [12], in a retrospective review of 115 donors, also found a weak correlation between split renal volume (ellipsoid method) and SRF by nuclear renography (*R* = 0.22–0.28), regardless of the technician who evaluated the volumes. Barbas et al. [22] used automated software to measure parenchyma volume through 3D reconstruction volume calculation, showing only a modest correlation compared to nuclear renography with Tc99m-DTPA (left kidney *R*^2^ = 26.2%, right kidney *R*^2^ = 26.7%). In addition, an acceptable clinical agreement between techniques was shown in the Bland–Altman analysis (Fig. 2), with 95% limits of agreement from − 8.51 to 6.11%. The mean difference is − 1.2%. On average, nuclear renography measures 1.2% more than CT scan for SRF of the remaining kidney, as expected in a population where nuclear renography was the main factor for the decision on the side of nephrectomy.

To evaluate the predictive ability of each imaging technique for remaining kidney function, we used eGFR based on serum creatinine levels because it is feasible and the most common method worldwide [9]. In epidemiological studies, the CKD-EPI equation has been shown to perform a more pertinent CKD diagnosis and staging [23], and most published studies have used it. We presented the results of eGFR using the CG-BSA-adjusted in addition to the CKD-EPI formula, as none was validated in a living donor population and CG is representative of 24 h creatinine clearance, which remains an important metric in the living donor evaluation [9], a not very heterogeneous population with median weights. In our study, both imaging techniques correlated with eGFR at one year, although CT volume showed a better correlation than nuclear renography for both formulas (Fig. 3). The results were similar in the multivariable linear regression analysis of renal donor function at 1-year post-donation, according to each imaging modality prediction adjusted for age, sex, laterality of the remaining kidney, and BMI (Table 3). Surprisingly, the best correlation was found with eGFR using the modified CG-BSA formula for both imaging techniques. Although it is the only formula that considers donor weight, we adjusted it for BSA. In agreement with our results, Rule et al. [24] found a better correlation between eGFR using the CG equation and iodothalamate GFR (*R* = 0.35) than between eGFR using the MDRD equation and iodothalamate GFR (*R* = 0.26) in potential kidney donors. Halleck et al.[10] also described a better correlation between DTPA clearance and eGFR using the CG equation (*R* = 0.55) than using the MDRD equation (*R* = 0.37) or CKD-EPI equation (*R* = 0.30) in a population of living donors, arguing that all their donors had an excellent GFR > 80 ml/min. In contrast, both formulas have been validated in cohorts of patients with chronic kidney disease. Wahba et al.[19] also reported slightly higher correlations when CG was used instead of CKD-EPI.

The actual eGFR after donor nephrectomy was higher than the predicted renal function measured by each imaging technique. After nephrectomy, compensatory hypertrophy of the contralateral kidney is expected, and by three months, remaining kidney clearance increases to a mean GFR of approximately 65–75% of pre-donation renal function [9]. A recent study on glomerular hemodynamics after kidney donation noted that adaptive hyperfiltration after donor nephrectomy is attributable to hyperperfusion and hypertrophy of the remaining glomeruli without glomerular hypertension in most donors 6–8 years after donation [25]. Nevertheless, there is a discussion that adaptive hyperfiltration might result in faster progression of kidney disease, namely in certain groups of donors with a less functional reserve. Developing reproductive metrics to identify these patients is a significant challenge. The risk of ESRD in living donors is low, although an increased risk, compared to healthy controls, has been evidenced in two long-term studies [5, 6]. Because ESRD is rare, different groups have pursued several surrogates to improve living-donor selection and donor safety. Massie et al. [26] reported an independent association between the living kidney donor eGFR at postoperative six months and subsequent ESRD.

Nevertheless, no significant association was found with preoperative eGFR. One year post-donation, eGFR was assessed in this study as a surrogate for long-term renal function in the donor instead of earlier values after donation, as it represents the time point at which the mechanisms of compensatory hyperfiltration have almost stabilized and renal function reaches a more stable value, [3, 27]. However, Lam et al. [28] recently described that the plateau in living kidney donors is reached by five years after donation, which is consistent with the observations in our cohort.

The CT scan SFR performed better in our population for this task, considering both univariate and multivariate analyses and non-nested modeling tests. Barbas et al. [22] also showed a better predictive value of volumetry than nuclear renography for postoperative remaining renal function at six months in the donor Eum et al. [13], in a very recent study, also found that CT volumetry outperformed nuclear renography for predicting kidney function at all time points after donation (1, 6 months, and > 1 year), considering both concordant and discordant groups.

Other clinical implications of CT scan volumetry results have already been assessed by our group. The remaining kidney volume indexed to weight (RKV/W) was found to be a strong predictor of estimated glomerular filtration rate at 1 year and mid-term renal function after living-donor nephrectomy [29]. Considering recipients´ perspectives, we showed that lower donated kidney volume was associated with an increased risk of lower graft function one year after living donor transplant and suggested it can be a tool for better selection of donors to improve graft outcomes, particularly in the setting of multiple potential living donors or kidney paired exchange programs [30].

Our study has some limitations. First, donors were evaluated retrospectively, with only selected living donor candidates being assessed, which does not allow inference of the results for the global population of potential living donors, as those with significant SRF differences from nuclear renography were excluded from donation. Second, our cohort consisted only of Caucasian patients. In addition, eGFR using estimation equations to assess kidney function has limitations. In addition, an added value of our study cohort is its larger size and that all donors have been evaluated by both imaging techniques and have eGFR at one year.

Hence, we conclude that CT scan volumetry has a better predictive performance for one year of remaining renal function than standard nuclear renography. When there was disagreement between techniques versus cases in agreement, a higher SRF by nuclear renography was significantly associated with worse eGFR in the donor one year after donation, while a higher SRF on CT scan was not. One can draw a hypothesis that renal volume is a surrogate of the renal reserve in healthy individuals, but it must be tested. This study supports the use of evaluation of SRF by CT volumetry at our center, avoiding the need for nuclear renography, which could also translate into an improved living-donor experience and reduced costs to the healthcare system.

## Supplementary Information

Below is the link to the electronic supplementary material.

Supplementary file1 (DOCX 16 kb)

## Data Availability

Individual-level data were de-identified and will be provided upon justified request.

## Abbreviations

ACR: Acute cellular rejection
BMI: Body mass index
BSA: Body surface area
GFR: Glomerular filtration rate
CG: Cockcroft-Gault
CKD-EPI: Chronic kidney disease epidemiology collaboration
CT: Computerized tomography
CrC: Creatinine clearance
DTPA: Diethylenetriamine-pentaacetate
eGFR: Estimated glomerular filtration rate
ESRD: End-stage renal disease
GFR: Glomerular filtration rate
HR: Hazard ratio
IQR: Interquartile range
MAG-3: Mercapto-acetyltriglycine
MDRD: Modification of diet in renal disease
RRT: Renal replacement therapy
SD: Standard deviation
SRF: Split renal function
