# Supplementary material for: CT volumetry performs better than nuclear renography in predicting estimated renal function one year after living donation
Source: Int Urol Nephrol. 2022 Dec 24;55(3):553–62. doi: 10.1007/s11255-022-03441-9 (PMC9958147; doi:10.1007/s11255-022-03441-9)
Supplement: Supplementary file 1 — Supplementary file1 (DOCX 16 kb) [file 11255_2022_3441_MOESM1_ESM.docx]

Supplemental Material

Supplemental Table 1. Agreement between techniques of SRF with right kidney remaining

| Right kidney remaining | | | | |
| --- | --- | --- | --- | --- |
| Nuclear renography: difference in split function between right and left kidneys (R–L) | CT volumetry: difference in split function between right and left kidneys (R–L) | | | |
|  | <-5% | -5 to 5% | >5% | Total |
| <-5% | 10 | 12 | 1 | 23 |
| -5% to 5% | 21 | 63 | 7 | 91 |
| >5% | 8 | 19 | 17 | 44 |
| Total | 39 | 94 | 25 | 158 |

Observed full agreement: 90 (57%)

Weighted agreement: 76%, Expected agreement (to weighted agreement): 67%, Kappa: 0.272

Supplemental Table 2. Agreement between techniques of SRF with left kidney remaining

| Left kidney remaining | | | | |
| --- | --- | --- | --- | --- |
| Nuclear renography: difference in split function between right and left kidneys (R–L) | CT volumetry: difference in split function between right and left kidneys (R–L) | | | |
|  | <-5% | -5 to 5% | >5% | Total |
| <-5% | 6 | 7 | 1 | 14 |
| -5% to 5% | 7 | 9 | 2 | 18 |
| >5% | 0 | 2 | 1 | 3 |
| Total | 13 | 18 | 4 | 35 |

Observed full agreement: 16 (46%)

Weighted agreement: 71%, Expected agreement (to weighted agreement): 67%, Kappa: 0.127

Supplemental Table 3. Non-nested modeling comparing nuclear and CT scans to predict renal donor function at 1-year post-donation

|  | Competing models:  Model 1: nuclear scan  Model 2: CT volume | | | | |
| --- | --- | --- | --- | --- | --- |
| Formula used | Test | Hypothesis tested | P | Test outcome: Reject null hypothesis? | Interpretation |
| CKD-EPI | J-test | Null: Model 1 superior | <0.001 | Yes | CT volumetry superior |
|  |  | Null: Model 2 superior | 0.299 | No | CT volumetry superior |
|  | Cox-Pesaran test | Null: Model 1 superior | <0.001 | Yes | CT volumetry superior |
|  |  | Null: Model 2 superior | 0.131 | No | CT volumetry superior |
| CG | J-test | Null: Model 1 superior | 0.002 | Yes | CT volumetry superior |
|  |  | Null: Model 2 superior | 0.151 | No | CT volumetry superior |
|  | Cox-Pesaran test | Null: Model 1 superior | <0.001 | Yes | CT volumetry superior |
|  |  | Null: Model 2 superior | 0.060 | No | CT volumetry superior |

CT – computed tomography

CG - Cockcroft-Gault

CKD-EPI - Chronic Kidney Disease Epidemiology Collaboration
